# How do different goals affect students’ internal collaboration script configurations? Results of an epistemic network analysis study

**DOI:** 10.3389/fpsyg.2024.1410152

**Published:** 2024-09-09

**Authors:** Tugce Özbek, Martin Greisel, Christina Wekerle, Andreas Gegenfurtner, Ingo Kollar

**Affiliations:** ^1^Department of Educational Psychology, University of Augsburg, Augsburg, Germany; ^2^Department of Methods in Learning Research, University of Augsburg, Augsburg, Germany

**Keywords:** computer-supported collaborative learning, script theory of guidance, achievement goals, epistemic network analysis, higher education

## Abstract

**Introduction:**

Research has suggested that how learners act in CSCL environments is considerably influenced by their internal collaboration scripts. These scripts are knowledge structures that reside in an individual’s memory and consist of play, scene, scriptlet, and role components. In its “internal script configuration principle,” the Script Theory of Guidance suggests that as learners work in a CSCL environment, these components are dynamically (re-)configured, and that this (re-)configuration is influenced by the goals of the individual learner. However, this principle has not yet been tested empirically.

**Methods:**

In this study, upon entering a CSCL environment, we therefore experimentally manipulated the goals that students pursued while learning. In one condition, we induced learning goals while in the other condition, no goals were induced. A total of 233 pre-service teachers collaborated in dyads on the task to analyze an authentic, problematic classroom situation by aid of educational evidence. We measured their internal scripts both at pre-test (i.e., before collaboration and before goal induction) and post-test (i.e., after collaboration and goal induction), focusing on the scriptlet level.

**Results:**

Results show that goal induction had no effects on the kinds of scriptlets participants selected during collaboration. However, results from Epistemic Network Analysis show that learning goal induction led to significantly different combinations of scriptlets (especially to more relations between scriptlets that are indicative of pursuing learning goals) than no goal induction. Furthermore, participants from the learning goal induction acquired significantly more knowledge about educational theories and evidence than students from the control condition.

**Conclusion:**

This study is among the first to provide direct evidence for the internal script configuration principle and demonstrates the effectiveness of inducing learning goals as a scaffold to support students’ knowledge acquisition processes in CSCL.

## 1 Introduction

A vast amount of research over the past 30 years has demonstrated that computer-supported collaborative learning (CSCL) can be a powerful method to facilitate learning in different educational contexts (Chen et al., 2018; Radkowitsch et al., 2020; Stahl and Hakkarainen, 2021; Vogel et al., 2017; Yang, 2023). During CSCL, learners are encouraged to actively participate in the learning process through negotiating and discussing ideas with peers (Roberts, 2005). That this really is the case is corroborated by meta-analytic findings that demonstrate the effectiveness of CSCL across a broad range of disciplines (e.g., Jeong et al., 2019; Xu et al., 2023) and digital technologies (e.g., Sung et al., 2017), yielding positive effects of CSCL both on domain-specific knowledge and cross-domain competences (for an overview, see Kollar et al., 2024).

The way learners actually learn and collaborate in CSCL environments can be considered as being influenced by two factors that mutually interact with each other: (a) the design of the learning environment and (b) the individual group members’ learning prerequisites. With respect to (a), research on CSCL has accumulated a considerable number of insights (Miller and Hadwin, 2015; Vogel et al., 2017), for example on the effects of different kinds of scaffolds on the quality of learning processes and outcomes. Yet, with respect to (b), empirical research seems to be scarce (Hsu et al., 2008; Prinsen et al., 2007). In this article, we therefore focus especially on this latter topic.

One prerequisite that has received attention in CSCL research is the learners’ internal collaboration script (Kollar et al., 2006). Internal collaboration scripts are cognitive structures of individuals that guide them in the way they understand and act in collaborative learning situations (Fischer et al., 2013). They are assumed to consist of different knowledge components (play, scenes, roles, and scriptlets) that are dynamically configured in learners’ memory. This assumption has been formulated in the Script Theory of Guidance (SToG) by Fischer et al. (2013), which also is concerned with how external scripts (scaffolds that structure a group’s collaboration process through specifying, sequencing, and distributing learning activities and roles among its members) affect collaboration and learning. In total, the SToG proposes a total of seven principles that describe the roles of internal and external scripts during CSCL. With respect to learners’ internal scripts, one central principle of the SToG refers to the assumption that the configuration of internal script components that learners (typically unconsciously) select to make sense of a given CSCL situation is influenced by the current goals of the learners (internal script configuration principle).

Surprisingly though, this principle has hardly been tested empirically so far. In fact, learners can have very different goals when collaborating, and it is not clear how exactly these different goals affect the configuration of their internal collaboration scripts and subsequent knowledge acquisition (Pintrich, 2000a). Following a well-known distinction from Achievement Goal Theory (Dweck and Leggett, 1988; Elliot, 2005), some learners may engage in learning and studying with the goal to arrive at a deep understanding of the learning material (learning goal). Other learners, in contrast, may rather engage in learning and studying because they pursue the goal to demonstrate how competent they are (performance goal). For this reason, in this paper, we investigate how different goals, respectively their induction, are related to learners’ internal script configuration and knowledge acquisition in the context of CSCL.

## 2 Literature review

### 2.1 Components of learners’ internal collaboration scripts in CSCL according to the script theory of guidance

The term CSCL covers many different instructional methods, all of which have in common that “peers interact […] with each other for the purpose of learning and with the support of information and communication technologies” (Suthers and Seel, 2012, p. 1). Based on recent meta-analyses, CSCL offers a vast potential to support learners’ academic achievement, for example, because it promotes students’ knowledge acquisition or skill development (Chen et al., 2018). From a theoretical point of view, there are many potential benefits of collaborative learning, even without the support of digital technologies, which include academic (e.g., fostering knowledge acquisition and critical thinking), social (e.g., developing social skills) or affective-motivational aspects (e.g., reducing anxiety; see Johnson and Johnson, 1989; Laal and Ghodsi, 2012).

However, there may also occur problems during collaborative learning, such as individual learners contributing little to collaboration or learners not actually working together but rather splitting the tasks among themselves (Roberts, 2005; Salomon, 1992). Especially under such circumstances, the collaborative learning process may be supported by the use of digital technologies, for example by providing tools to organize learners’ ideas and contributions, to provide resources, or guidance to structure the collaboration process (Stahl et al., 2006; Suthers and Seel, 2012).

A theoretical approach that conceptualizes such guidance is the Script Theory of Guidance (SToG; Fischer et al., 2013), which focuses on one particular kind of guidance that has received very much attention in CSCL research: so-called “external collaboration scripts.” External collaboration scripts provide group learners with guidance on the kinds and sequence of activities and roles they are supposed to take over during collaboration, often supporting their execution via prompts or sentence starters. SToG, however, assumes that what actually happens during collaboration is not only influenced by such external collaboration scripts, but also by the learners’ internal collaboration scripts.

**Figure 1:**
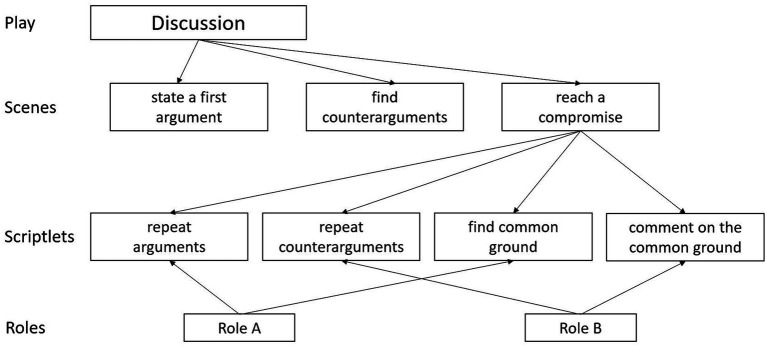
Example of an internal collaboration script of a learner during a discussion. Only a few examples of possible scriptlets are shown. Theoretically, every scene is connected to a specific set of scriptlets.

According to the SToG, internal collaboration scripts are configurations of knowledge components in the learner’s cognitive system that determine their understanding and acting in a given collaborative situation. Based on Schank’s (1999) dynamic memory approach, a basic tenet of the SToG is that internal scripts consist of configurations of four different kinds of script components (see Figure 1): (a) The “play” component includes knowledge about the kind of the situation an individual experiences, e.g., a discussion held in a chat forum or the joint writing of a blog post. Once a learner has (consciously or unconsciously) selected a specific “play,” this “play” then connects a set of (b) “scenes.” Scenes refer to the person’s knowledge about the different situations that typically make up the play. In a discussion, for example, a learner’s “discussion play” might include a scene in which the group collects information to develop arguments, while another scene might be to exchange arguments. Once a certain scene is activated, the person also has expectations on what kinds of activities are typically part of that scene. Knowledge about the kinds and sequence of the activities that are likely to occur during that scene, are then represented in so-called (c) “scriptlets.” Therefore, scriptlet components describe the learners’ knowledge about sequences of activities within particular scenes (Schank, 1999). In the scene “reaching a compromise,” for an exemplary learner, the first scriptlet might refer to the activity “summarize the most important arguments,” while for another learner whose internal script might include the same scene, the first scriptlet in that scene might refer to the activity “define what would count as compromise.” Finally, learners hold knowledge about different kinds of (d) “roles,” i.e., about the question what kinds of activities are likely to be taken over by what person in the given collaborative situation. Similar to a theatre play, roles may extend over several scenes and include several activities.

### 2.2 The internal script configuration principle within the script theory of guidance

A central assumption of the SToG is that through experience, learners acquire a range of different plays, scenes, scriptlets, and roles, and that these knowledge components are dynamically combined in each new situation, depending on the individual’s perception of the current situation, and on the goals they pursue in that situation. This idea lays the foundation of the so-called configuration principle of the theory. Literally, this principle states that “How an internal collaboration script is dynamically configured by a learner from the available components to guide the processing of a given situation, is influenced by the learner’s set of goals and by perceived situational characteristics” (Fischer et al., 2013, pp. 57–58).

As described, the internal collaboration script consists of different knowledge components that refer to collaborative learning situations that are considered. These components are assumed to be very flexible in the way they are combined with each other. This means that in any situation, a learner may select different plays, scenes, scriptlets, and roles available in memory that – from their subjective perspective – are promising to make sense of the current situation. Even small changes in the situation and changing requirements can result in a quick (and very often subconscious) adaptation and reconfiguration of the internal collaboration script components. For example, certain tool features such as a flashing cursor might indicate an opportunity to enter a text, making learners’ selection of a scriptlet “enter text” more likely than if there was no such flashing cursor (Fischer et al., 2013; Kirschner et al., 2008; Schank, 1999).

Yet, not only situational characteristics (i.e., external factors) may influence a learner’s internal script configuration, but also personal characteristics may do so (i.e., internal factors). As formulated in the internal script configuration principle, this refers in particular to learners’ goals. Thus, learners’ current set of goals can, on the one hand, influence the *selection* of script components, i.e., plays, scenes, scriptlets, and roles. This means that the learner is likely to choose or act out those script components that appear as most useful to pursue their current goals, resulting in an engagement in activities that are conducive to reaching those goals. Simultaneously, scriptlets that refer to activities that are not in line with the current goals are inhibited. For example, if a learner notices that their learning partner does not seem to exert effort during collaboration, and if they have the goal to get the task done anyway, they may de-activate scriptlets that would guide them to ask their learning partner for input and replace this scriptlet by a scriptlet “solve the task alone.” On the other hand, goals may also influence the order of the scriptlets of a learner’s internal script (i.e., how these different activities are *sequenced*). For example, if a learner’s dominant goal is to do well on a subsequent multiple-choice exam, they might first select a scriptlet “solve items of the test exam,” and only later select scriptlets that might help them reach a deeper understanding of the learning material (e.g., “discuss how the different concepts relate to each other” or “jointly develop examples for the application of the information.” In contrast, a learner who is in the same situation, but whose primary goal is to arrive at a deep understanding of the learning material might select the reverse order of scriptlets and start with more deep-level learning activities and use the scriptlet “solve items of the test exam” only afterwards.

As Fischer et al. (2013) point out, a study by Pfister and Oehl (2009) provides (indirect) evidence for the influence of goals on the configuration of learners’ internal collaboration scripts. This study investigated how goal focus, task type, and group size influence synchronous net-based collaborative learning discussions. For this purpose, they varied the goal focus of the learners: in one condition, participants should take on an individual focus (i.e., they received rewards based on their individual performance), while in a second condition, they should take on a group focus (i.e., they received rewards based on their group’s performance). Results indicated that learners with the group focus used more supporting functionalities of the tool (e.g., the possibility to mark what previous chat message one’s own message refers to) than learners with the individual focus. Fischer et al. (2013) interpret this finding in a way that the different foci of the learners led to a (re-)configuration in their internal scripts, as represented in learners’ use of different tool functions.

However, a couple of limitations of this study and of Fischer et al.’s (2013) interpretation need to be noted here. First, the (re-)configuration of the internal script is only inferred indirectly (from the use of a certain feature by the learners), rather than measured directly. To do that, it would be necessary to apply methods that indicate the kinds of internal script components and provide insight into their sequential nature. Second, the authors of the study did not examine how the internal script of the learners was structured. Therefore, it is also not possible to assess to what extent this script and its components actually reconfigured during collaboration. Third, “goal focus” was experimentally manipulated by providing rewards either for individual or for collaborative performance. In addition, the SToG does not offer an in-depth conceptualization of the concept of learners’ goals (Stegmann et al., 2016). While a “goal focus” is certainly one way to think about goals, research on Achievement Goal Theory (e.g., Elliot and Fryer, 2008) has focused on goals that have been shown to have even tighter relations to learning processes and outcomes. This research will be discussed in more detail in the next section.

### 2.3 Learners’ goals during CSCL

Goals influence students’ learning processes. From a theoretical perspective, they describe a standard by which learners can assess their learning progress and initiate regulatory processes accordingly (Pintrich, 2000b). Goals that a learner pursues in learning and performance contexts are referred to as achievement goals. They describe the reasons why learners engage in competence-related behavior (Elliot, 2005; Elliot and Fryer, 2008). Even though more nuanced goal typologies have developed over the years (e.g., Bardach et al., 2022), at a global level, achievement goal theory distinguishes between two types of goals. Firstly, in a given situation, learners may pursue so-called *learning goals*, which means that learners engage in learning because they are motivated to improve their competence. Secondly, they may also have *performance goals*; for learners with these goals, it is particularly important to engage in learning in order to demonstrate their performance or to outperform others (Heyman and Dweck, 1992). With respect to the dichotomy of learning goals and performance goals, empirical research has often shown positive relations between learning goals with various favorable learning processes and outcomes (Hulleman et al., 2010; Payne et al., 2007). For example, students who pursue learning goals have been reported to be likely to persist on difficult tasks, to use deep level instead of surface level learning strategies, and (though not consistently, see Darnon et al., 2007) reach high levels of achievement (Meece et al., 2006). When learners pursue performance goals, in contrast, they typically rather use surface-level learning strategies (Payne et al., 2007), which in turn can be helpful to solve easy academic tasks but can be detrimental when more difficult tasks are posed (Utman, 1997). These findings indicate that different achievement goals may lead to different learning processes and outcomes.

In terms of CSCL, research also investigated strategies specifically using social interaction as means to learning. It found that the more students have mastery goals, the more they tend to seek help (Karabenick and Gonida, 2018; Ryan and Pintrich, 1997) and ask for feedback (Cellar et al., 2011; Payne et al., 2007; VandeWalle and Cummings, 1997). Students with strong mastery goals also prefer other students with mastery goals as teammates (Barrera and Schuster, 2018). Therefore, we can expect students to engage the more in regulating collaborative learning, the more they pursue mastery goals. And indeed, Greisel et al. (2023) found that students intended to execute more different strategies directed at the self-, co-, and socially shared level during collaborative learning, the more mastery goals they had. In contrast, performance goals were not related to help-seeking and asking for feedback (Cellar et al., 2011; Payne et al., 2007; Senko and Dawson, 2017). Instead, students with high performance goals engage more in self-handicapping (Senko and Dawson, 2017), and feedback seems to diminish their performance (VandeWalle et al., 2001). Thus, they seem to be more concerned with their impression than their learning in social circumstances. However, in small collaborative learning groups at least, they do intend to engage in socially shared regulation but not co-regulation (Greisel et al., 2023). Furthermore, a study by Giel et al. (2021) also suggests that the compatibility of learners’ achievement goals affects collaborative learning outcomes. For example, the results indicate that the degree of compatibility of group members’ mastery goals is linked to engagement, whereas the degree of incompatibility is related to performance, highlighting the importance of examining students’ achievement goals in collaborative learning processes (Giel et al., 2021). From these findings, we conclude that different achievement goals prepare students for different behaviors during collaborative learning.

At a cognitive level and in the context of the SToG, these differences should be represented by differences in the configuration of learners’ internal collaboration script, i.e., the knowledge they activate regarding the kinds and sequences of activities that are likely to occur during collaboration. While hypothetically, learners have different internal script components (in this case, scriptlets) available in their dynamic memory that would enable them to engage in the collaboration process, they (consciously or unconsciously) select those scriptlets that fit their personal goals the best and disregard those that do not (Stegmann et al., 2016). For example, a pronounced learning goal might encourage learners in a collaborative situation to select scriptlets that lead them to ask more questions or ask for feedback from their peers. Thus, these learners could select scriptlets leading them to engage more actively in the collaboration process in order to acquire more knowledge, and omit or postpone others, for example, the scriplet “agree with learning partner.” In contrast, learners with pronounced performance goals might be more likely to select scriptlets that are related to impression management techniques as they might want to only appear competent, for example by using subject-specific language very deliberately (Greisel et al., 2023). In turn, they might, for instance, omit the scriptlet “ask for feedback,” in order to avoid being perceived as incompetent. This, in turn, might also imply that (the induction of) different achievement goals within CSCL could influence students’ knowledge acquisition. Yet, empirical evidence on these issues seems to be missing so far.

### 2.4 Research questions and hypotheses

In sum, evidence from the field of CSCL, but also from research on achievement goals, indicates that different goals of learners may influence how learners act in collaborative learning. On this basis, the SToG argues that learners’ goals in CSCL lead to specific configurations of different script components (plays, scenes, scriptlets, roles) in the learners’ cognitive systems, both with respect to the kinds of scriptlets that are selected, and with respect to the sequential organization in which they are combined with each other. However, so far there has been little research investigating this configuration and the actual change in the internal script as a function of the presence or absence of different achievement goals. Moreover, to date, there is a scarcity of research examining the impact of such goals on knowledge acquisition within the context of computer-supported collaborative learning. Therefore, in the present study, we actively manipulated learners’ goals either in the direction of an actualization of learning goals or of performance goals or no goal induction and investigated the effects of this manipulation on their internal collaboration scripts (more precisely, the scriptlets) when working on a CSCL task.

Our first research question was: Does the induction of different kinds of achievement goals (no induction vs. learning goals induction vs. performance goals induction) influence the configuration of the internal collaboration script? We hypothesized (H1) that learners from the three conditions would select different scriptlets to guide their collaboration. Furthermore, we assumed that not only the selection, but also the sequential organization of scriptlets would differ depending on whether or what kind of an achievement goal is induced (H2).

Additionally, to date, there is only limited research on how prompting specific achievement goals during collaborative learning within the tool influences students’ knowledge acquisition. Thus, we wondered whether the induction of different goals would affect the knowledge students would acquire as an outcome of their collaboration. For this reason, our second research question was: Does the induction of learning vs. performance goals vs. no goals affect students’ knowledge acquisition in a collaborative task? Based on research on achievement goals (e.g., Hulleman et al., 2010; Meece et al., 2006; Payne et al., 2007), we hypothesized that the induction of learning goals would lead to a higher knowledge acquisition than when no goals would be induced (H3). Since previous research on performance goals indicates a relatively mixed picture, we only anticipated that the performance goal condition would differ from the control condition (H4) regarding students’ knowledge acquisition.

## 3 Method

### 3.1 Participants and design

A total of *N* = 233 pre-service teachers participated in the study. They were on average 22 years old (*M*_Age_ = 22.3, *SD*_Age_ = 2.3), mostly female (72%) and in their fifth semester (*M*_Sem_ = 5.0, *SD*_Sem_ = 1.1). They were enrolled in a teacher education program for elementary school teachers, middle school teachers, high school teachers, and secondary school teachers of various subjects. The study was embedded as a compulsory part of a course in educational psychology for pre-service teachers. Their task was to analyze an authentic, written case that described a problematic classroom situation and a teacher’s efforts to solve those problems. All students within these courses were invited to participate in the additional scientific data collection. However, the students were free to decide whether they wanted to participate and suffered no disadvantages if they decided against it. Students who decided to participate received no reward for their participation. The potentially available sample was therefore determined by the sizes of the courses that were available for data collection.

We conducted a sensitivity analysis with G*Power [3.1.9.7] to assess the effect size that was realistically detectable with our sample size. Since one group was later excluded (see Section 4.1) only two groups remained with 71 and 72 students, respectively. We calculated the sensitivity analysis with an α-error probability set to 0.05 and desired power set to 0.80. For an ANOVA with one between-subject factor with two levels and one repeated measurement with two time points, the analysis revealed that an interaction effect with Cohen’s *f* = 0.12 could be detected, indicating a sufficient likelihood to detect small to moderate interaction effects. For a t-test comparing two independent groups, the analysis indicated that an effect size of Cohen’s *d* = 0.47 could be detected, indicating a sufficient likelihood to detect moderate effects.

Students worked on the task in the learning management system Stud.IP. To support their collaboration, they used a collaboration tool “coLearn!.” This tool serves to structure collaborative learning with external collaboration scripts by giving instructors the opportunity to assign, sequence, and distribute learning activities and roles, to specify prompts, and to provide tasks and learning materials. To investigate how different achievement goals impacted their internal collaboration script configuration, we established a 1 × 3 between-subjects design with the conditions “induction of learning goal,” “induction of performance goal,” and a control group (no goal induction). The conditions differed in that the instructions within coLearn! included prompts that highlighted the respective goal. More specifically, for example, participants in the learning goal conditions were told that completing the learning task would be very important in order to expand their skills. In contrast, participants in the performance goal condition were told that their performance would be evaluated by their instructor (see Table A1). Lastly, participants in the control condition did not receive any such information about the goals that were to be pursued. Participants were randomly assigned to dyads and to one of the three conditions mentioned before.

### 3.2 Procedure

**Figure 2:**
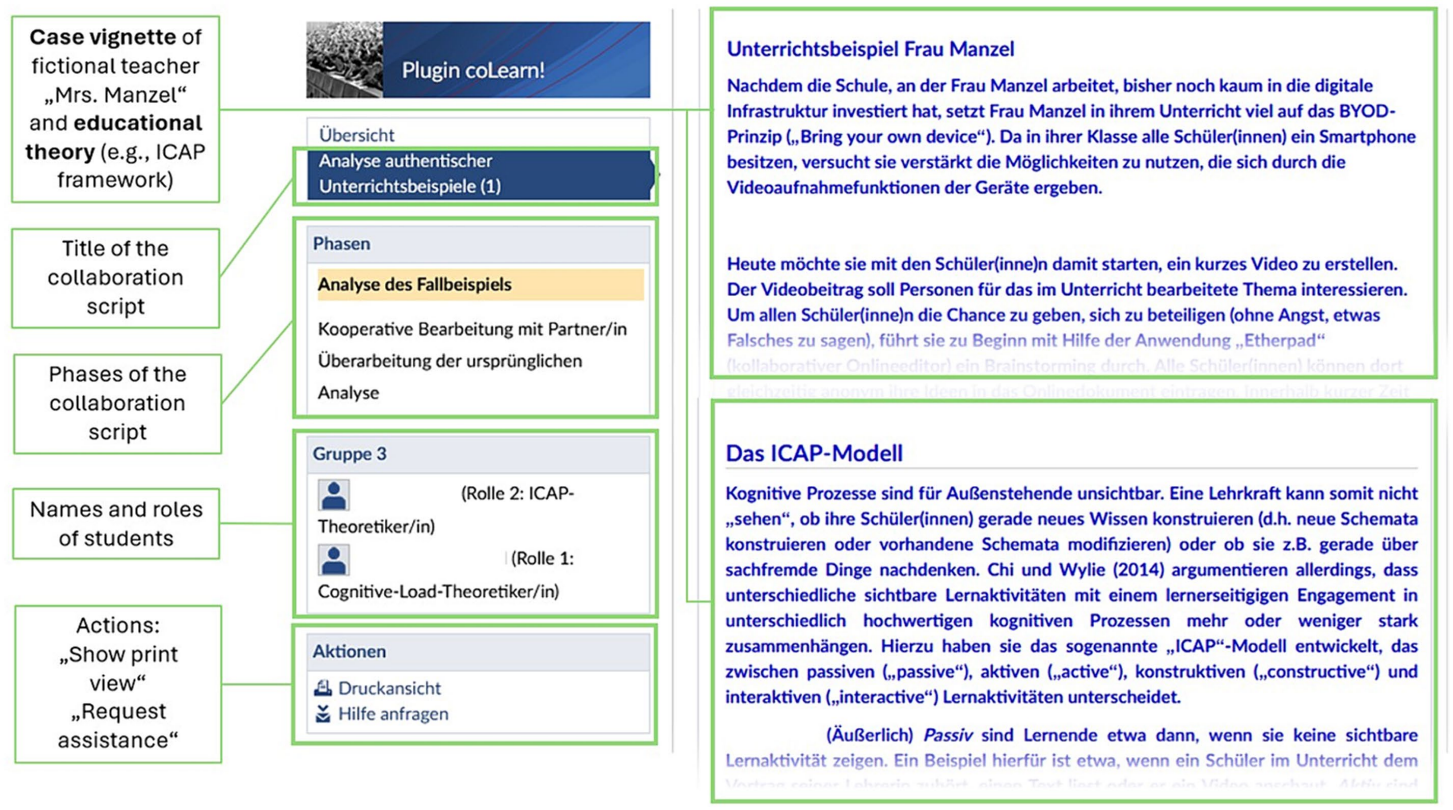
User interface of the tool “coLearn!”. In the right area, the case vignette, educational theories, and instructions were displayed. Depending on the specific role of each student, the tool provided either a text about the Cognitive Load Theory (Sweller, 2011) or the ICAP framework (Chi and Wylie, 2014).

During pretest, we measured students’ initial internal collaboration scripts and knowledge of the theoretical concepts that would be addressed during case analysis (i.e., the Cognitive Load Theory by Sweller, 2011, and the ICAP framework by Chi and Wylie, 2014). For three weeks, the students then worked in pairs using “coLearn!.” In week 1, they were instructed to individually analyze a case vignette that described several problematic teacher actions (e.g., prompting students to only watch other students recording a video without any other further instruction; prompting students to integrate items in their videos that are not conducive to the learning goals) during her lesson, using one of two scientific educational theories: Cognitive Load Theory (Sweller, 2011) or the ICAP framework (Chi and Wylie, 2014). The case vignette was 421 words long and did not contain any information on the lesson’s content (see Figure 2). The educational theories were presented as texts explaining central elements of the theories, supported by examples. The length of the texts was comparable (Cognitive Load Theory: 806 words, ICAP framework: 800 words). In week 2, these analyses were swapped between the students within a dyad, and the students were instructed to evaluate the analysis of their respective partner and to expand on it with the help of the respective other theory. In week 3, the students received the evaluation and elaboration from their peer and were asked to use it to revise their original analysis. After this three-week collaboration phase, we conducted a manipulation check by asking the participants whether, during the use of the tool, (1) it was particularly important to develop their competencies (learning goal), or (2) to achieve a high performance (performance goal). Moreover, the students’ internal collaboration scripts and knowledge about the theories were measured again.

### 3.3 Goal induction

As described, the two goal conditions differed from each other with respect to the presentation of statements in coLearn!. Each week, a new page with instructions and text fields was displayed in the tool. In addition to specific instructions regarding the case analysis that were identically presented in the control group, the prompts highlighted a specific goal. In the *learning goal* condition, after instruction, the task was labeled as “important in order to improve one’s own competencies and to successfully cope with problems in later professional life.” In addition, working with the tool was explicitly framed as a learning opportunity in this condition. In contrast, in the *performance goal condition*, it was stated that the task was “important in order to achieve good grades.” In addition, working with the tool was framed as an “important opportunity to prepare for exams,” and it was stated that tutors would check the assignments later (see Table A1).

### 3.4 Dependent variables

#### 3.4.1 Internal collaboration scripts

In order to assess the learners’ internal collaboration scripts, prior to using the collaboration tool, in the pretest participants described how they would generally carry out a collaborative analysis of a problematic classroom situation. Based on a synopsis of typical collaborative activities (Csanadi et al., 2018), they were given a list of 40 activities (e.g., “asking questions,” “reflecting on the theory” etc.) to choose from, representing different scriptlets that could be carried out as part of such a collaboration (see Table A2). The participants were instructed to drag and drop the activities they would perform from the list and put them in the order in which they would perform them. In this way, we captured their internal collaboration scripts regarding a collaborative case analysis considering the kinds of scriptlets and their specific sequence. In the posttest, participants were instructed to select and arrange activities from the same set of activities, again using drag-and-drop and arrange them according to how they actually carried them out during collaboration. Afterwards, we conducted an expert survey to categorize the activities that the students could select. For this purpose, nine experts from the field of educational psychology who are well-versed in the area of collaborative argumentation and, on average, had been working in related research for 4 years (*Mdn* = 3.5) were asked to categorize the activities. To do this, they should assess whether they would associate them with a learning goal orientation, performance goal orientation, both, or neither of them, in the context of conducting a collaborative case analysis. If over 50% of the experts indicated that a specific activity could clearly be assigned to one of the two goal orientations, that activity was categorized accordingly. In this way, 16 out of the total of 40 activities were classified as typically related to learning goals and 8 as typically related to performance goals (see Table A2). For the remaining activities, no clear consensus was reached, or the researchers assigned the activity either equally to both or none of the goals. Subsequently, for the pre- and the post-test separately, we calculated a total score for each participant by coding whether the students selected a specific activity (1) or not (0).

#### 3.4.2 Achievement goals

To ensure that there were no pre-existing differences in goal orientations between the groups, students were surveyed their goal orientations prior to the intervention (Daumiller et al., 2019). They were required to answer four items each to assess their learning goal orientation (e.g., “When using the plug-in, developing my competencies is particularly important to me,” Cronbach’s *α* = 0.92), and performance goal orientation (e.g., “When using the plug-in, achieving a good performance is particularly important to me,” Cronbach’s *α* = 0.80).

#### 3.4.3 Knowledge

During pre- and post-test, students were required to answer a total of 48 items in the form of multiple-choice questions to assess their content-specific knowledge about the theories that they were supposed to apply to the cases. They had to decide whether they agreed with each of the four options based on a preceding question and received a point for each correctly selected answer. The items were formulated based on the theoretical texts that the students were given to use during their case analysis, thus addressing aspects of the ICAP framework (Chi and Wylie, 2014; 20 items) and the Cognitive Load Theory (Sweller, 2011; 28 items). For instance, a question concerning the Cognitive Load Theory was, “What are potential sources of extraneous load during the processing of multimedia representations?.” The options for answers below included, for example, “Background music in animations” (correct) and “Prompts to self-explain the learning material” (incorrect). Based on the correctly answered questions, a percentage was calculated, allowing the students to achieve a total score between 0 and 1 in the pre- or posttest, respectively.

### 3.5 Statistical analyses

To test whether the configurations of participants’ internal collaboration scripts differed between the selected scriptlet categories (H1), we conducted a mixed ANOVA for the sum of scriptlets in each category as dependent variable. The condition represented the between-subjects factor (learning vs. performance vs. control), whereas time was used as within-subjects factor (pre- vs. posttest).

Regarding the potential difference in the sequential organization of scriptlets (H2), we conducted an Epistemic Network Analysis (ENA; Shaffer et al., 2016). ENA is an innovative approach for measuring, illustrating and understanding co-occurrences of activities in sequential data. It describes a body of techniques to detect and quantify relations between elements of coded data and visualizes them in network graphs, which display the structure and strength of connections between codes. For each student, the co-occurrences of their selected activities were accumulated by using a moving window that goes through the indicated activity sequences and determines, which activities in the sequence were occurring in the same temporal context and are consequently positioned at a close distance in the data (Shaffer et al., 2016). In the present analysis this window was set to 4 activities, meaning that the algorithm looked for co-occurrences between an activity and the three activities that preceded it. Since, overall, participants reported an average of 16 activities (*M* = 15.85, *SD* = 5.20) in the post-test, we chose a window size as small as possible which still allowed for meaningful co-occurrences of learning activities. As we aimed to compare students in different experimental groups, the analysis further accumulated co-occurrences of activities for students per experimental group as the unit of analysis. The resulting cumulative adjacency matrices are converted into adjacency vectors and normalized to transform frequencies of co-occurrences into relative frequencies. Using a dimensionality reduction approach (singular value decomposition), the original high-dimensional vector space is rotated to identify those dimensions that explain most variance in the data. The result is a multidimensional network model that can be depicted as two-dimensional network graphs.

In the two-dimensional network graphs, the activities are represented as gray nodes, with the relative size of the nodes indicating the relative frequencies of activities. The nodes are connected by colored lines (also referred to as edges), with the thickness of the lines representing the relative frequencies of co-occurrences of activities (i.e., relative strength of their sequential connection). Accordingly, instead of interpreting the absolute thickness of lines, it is relevant to compare the thickness of lines in comparison to the other lines. To facilitate comparisons between the different experimental groups, ENA can subtract one group’s network from the other group’s network, allowing for the identification of the most significant differences between two networks. The resulting difference in the two networks’ edges are then visualized as a comparison graph, which we will mainly focus on in our interpretation for the purpose of the group comparison. In the comparison graph, thicker lines signify larger disparities in the intensity of a connection, while thinner lines reveal smaller differences in connection strength. The color of each line designates which of the two group’s network possesses the stronger connection.

Additionally, ENA maps what is called a centroid of a network. It represents the network as a single point in the projection space. Similar to a center of mass of an object, the centroid of the network graph is constituted by the arithmetic mean of the edge weights of the network model that are allocated according to the network projection in space. Hence, the centroid position can be interpreted similar to a group mean: closer located centroids indicate similar networks of the two groups, whereas centroids that are located more distant represent rather different networks of the two groups.

The calculation of centroids also allows for the statistical comparison of multiple networks. For this purpose, the network model is rotated such that systematic variance in the groups’ differences is shifted to the one dimension in the network space, which is why the two group’s centroids are then aligned with the x-axis. This enables performing a t-test to evaluate whether there are significant differences between the two groups’ networks. Subsequently, in the case of significant differences, researchers can visually inspect the subtraction network (see above) to identify which connections differ between the two networks (Shaffer et al., 2016). Before the analysis, we dropped the four least frequent activities (*n* < 10 in pre- or posttest). Two further activities were dropped after the first analysis which were not connected to the rest of the network and represented outliers.

To assess whether students in different experimental conditions gained a different amount of knowledge between pre- and posttest, we conducted a mixed ANOVA (H3 and H4). Analogously to the first hypothesis, the condition (learning vs. performance vs. control) represented the between-subjects factor and time (pre- vs. posttest) represented the within-subjects factor.

## 4 Results

### 4.1 Preliminary analyses

In order to perform a manipulation check, we conducted an ANOVA to determine whether there were differences between the groups concerning the perception whether it was particularly important to develop their competencies or to achieve a high performance while using the collaboration tool. The manipulation check revealed that there were only significant differences as expected between the learning goal condition and the control group regarding the perception that it was particularly important to develop competencies when using the tool, *F*(2, 230) = 5.03, *p* < 0.001, *η*^2^ = 0.04. Thus, there were no significant differences between learning goal condition and performance goal condition or performance goal condition and control group. Also, there were no significant differences regarding the perception that it was important to achieve a high performance between the performance goal condition and control group or learning goal condition and performance goal condition, *F*(2, 230) = 1.27, *p* = 0.28. Therefore, in the following analysis, only the learning goal condition and the control condition will be compared.

To ensure that these differences do not reflect *a priori* differences in students’ achievement goals, we investigated their general goal orientations prior to the intervention. A *t*-test demonstrated that there were no significant differences in terms of learning goal orientation (*t*(152) = 0.68, *p* = 0.50) or performance goal orientation (*t*(152) = −0.60, *p* = 0.55) between the learning goal condition and the control condition.

### 4.2 RQ1: effects on goal induction on students’ internal script configuration

#### 4.2.1 H1: effects of goal induction on scriptlet selection

To test H1 regarding the selection of the scriptlets in the pre- and posttest, we conducted two mixed ANOVAs, one for each category of activities we had identified based on the expert ratings described above. There was no significant interaction between time of measurement and condition with regard to learning goal associated activities, *F*(1, 143) = 0.17, *p* = 0.68; *η*^2^ = 0.001, or performance goal associated activities, *F*(1, 143) = 0.32, *p* = 0.57; *η*^2^ = 0.002. Thus, there were no significant effects of condition on the kinds of activities (scriptlets) participants mentioned to have used during collaboration from pre- to posttest. However, there was a significant main effect of time regarding the learning goal associated activities, *F*(1, 143) = 122.51, *p* < 0.001, *η*^2^ = 0.46, indicating a significant decrease of the sum of reported learning goal associated activities between pre- and posttest, but not in terms of the performance goal associated activities, *F*(1, 143) = 0.000, *p* = 0.99, *η*^2^ < 0.01. There was no significant main effect of group, meaning that both groups did not differ significantly regarding the learning goal associated activities, *F*(1, 143) = 0.01, *p* = 93, *η*^2^ < 0.01, or performance goal associated activities, *F*(1, 143) = 0.006, *p* = 94, *η*^2^ < 0.01.

#### 4.2.2 H2: effects of goal induction on scriptlet configurations

To evaluate H2, we conducted an ENA to compare the networks of scriptlets in each condition in the posttest (learning vs. no goal induced). The mean centroid value for scriptlets in the epistemic network of the learning goal condition was significantly different from the mean centroid value in the network of the control condition, *t*(131.53) = 9.56, *p* < 0.001, Cohen’s *d* = 1.67, indicating that the true difference in means is not equal to 0. Consequently, the results indicate that there were significant differences with respect to the sequential organization of the scriptlets selected between participants from the learning goal induction condition and the control condition.

**Figure 3:**
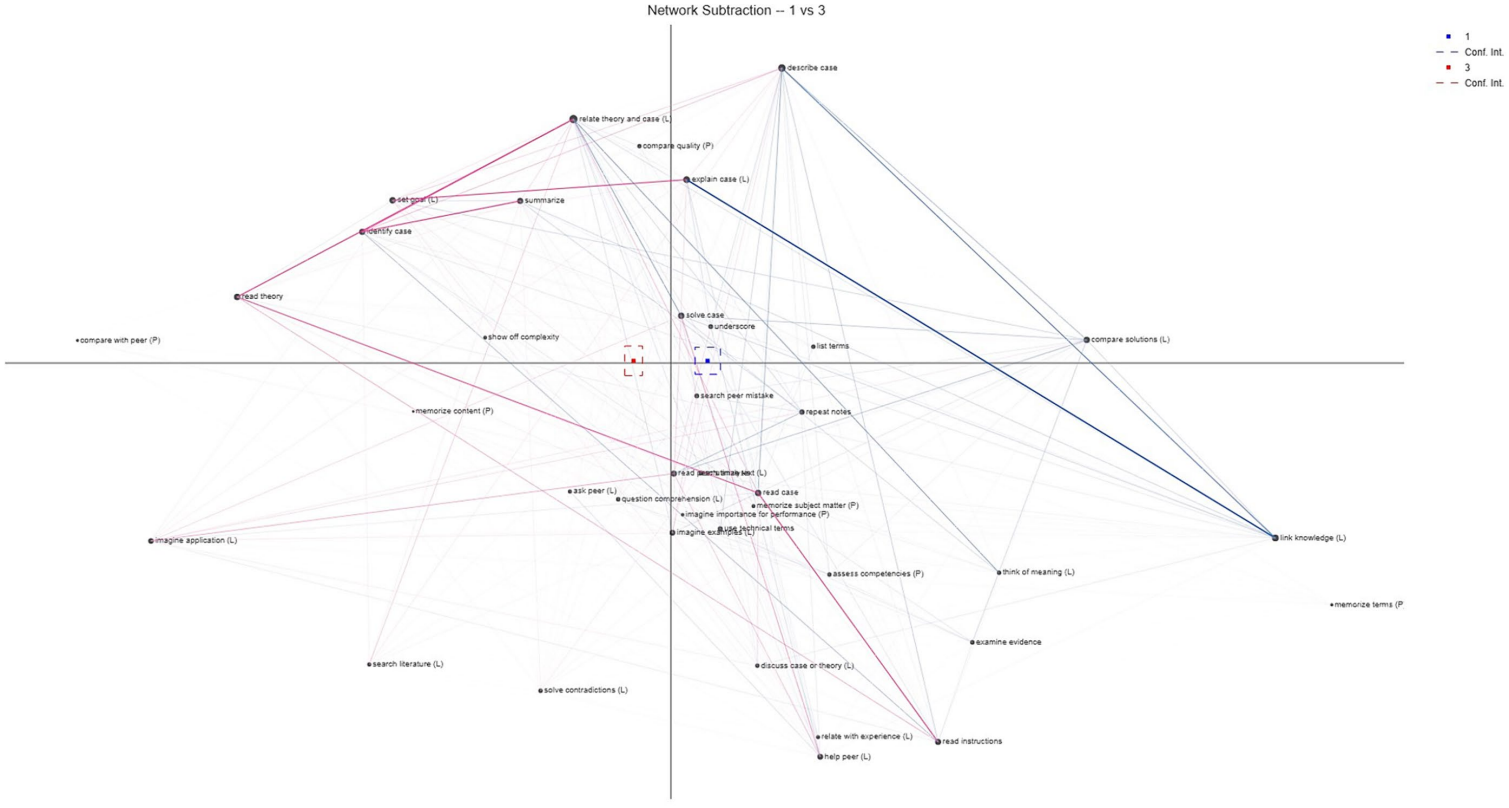
Epistemic network analysis of the difference between the networks of scriptlets in learning goal condition (1/blue) and control condition (2/red). Abbreviations following some activities represent their expert ratings (L = activities associated with learning goals, P = activities associated with performance goals).

Subtracting the networks (Figure 3) revealed that the network of the control condition (red), in comparison to the learning goal condition (blue), displayed stronger connections between the scriptlets “read theory,” “read case,” “identify problem” and “relate theory and case” as well as “summarize.” In addition, there were comparatively stronger connections between the scriptlets “set goal” and “explain case” as well as “read case” and “read instructions.” In contrast, the learning goal condition showed a much stronger connection particularly between the “explain case” and “link knowledge” scriptlets. There were also comparatively stronger connections between the scriptlets “describe case” and “link knowledge.” Overall, it thus appears that in the control condition, a variety of different activities were more strongly interconnected, whereas the learning goal condition showed stronger connections between a smaller number of activities.

To gain an exploratory understanding of how the activities could be evaluated in terms of the induced goals, we combined this analysis with the expert ratings by adding the abbreviation “(L)” for activities that, according to the expert ratings, are typically associated with learning goals, or “(P)” for activities that are typically associated with performance goals. First, it is important to note that the control condition did not involve any form of goal induction, meaning that no particular goal orientation was expected to emerge in this condition. Consequently, in theory, students can be expected to more or less equally engage in activities related to learning and performance goals. Furthermore, as described earlier, some activities could not be clearly attributed to either or any of the mentioned goals, which is why these activities were not annotated at all (see Figure 3). Additionally, overall, more activities were identified by the experts as associated with learning goals than with performance goals. However, it can be noted that, despite the overall lower number of strong connections in the learning goal condition, at least with regard to the link between the scriptlets “explain case” and “link knowledge,” two scriptlets associated with learning goals were strongly interconnected in this condition. In contrast, the scriptlets “read theory,” “read case,” “read instructions,” “identify case” and “summarize,” for which the results of the ENA show stronger connections in the control condition, could not be clearly associated with a specific goal by the experts. Yet, in the control condition, there also appeared strong connections between the scriptlets “set goal,” “relate theory and case,” and “explain case,” which are likewise associated with learning goals. Therefore, it could be summarized that in the control condition, numerous associations were observed, involving both learning and performance goal-related scriptlets, as well as scriptlets that could not be clearly categorized. In contrast, the learning goal condition appears to feature fewer associations, yet these seem to be predominantly linked to learning goal-related scriptlets.

### 4.3 RQ2: effects of goal induction on knowledge acquisition

**Figure 4:**
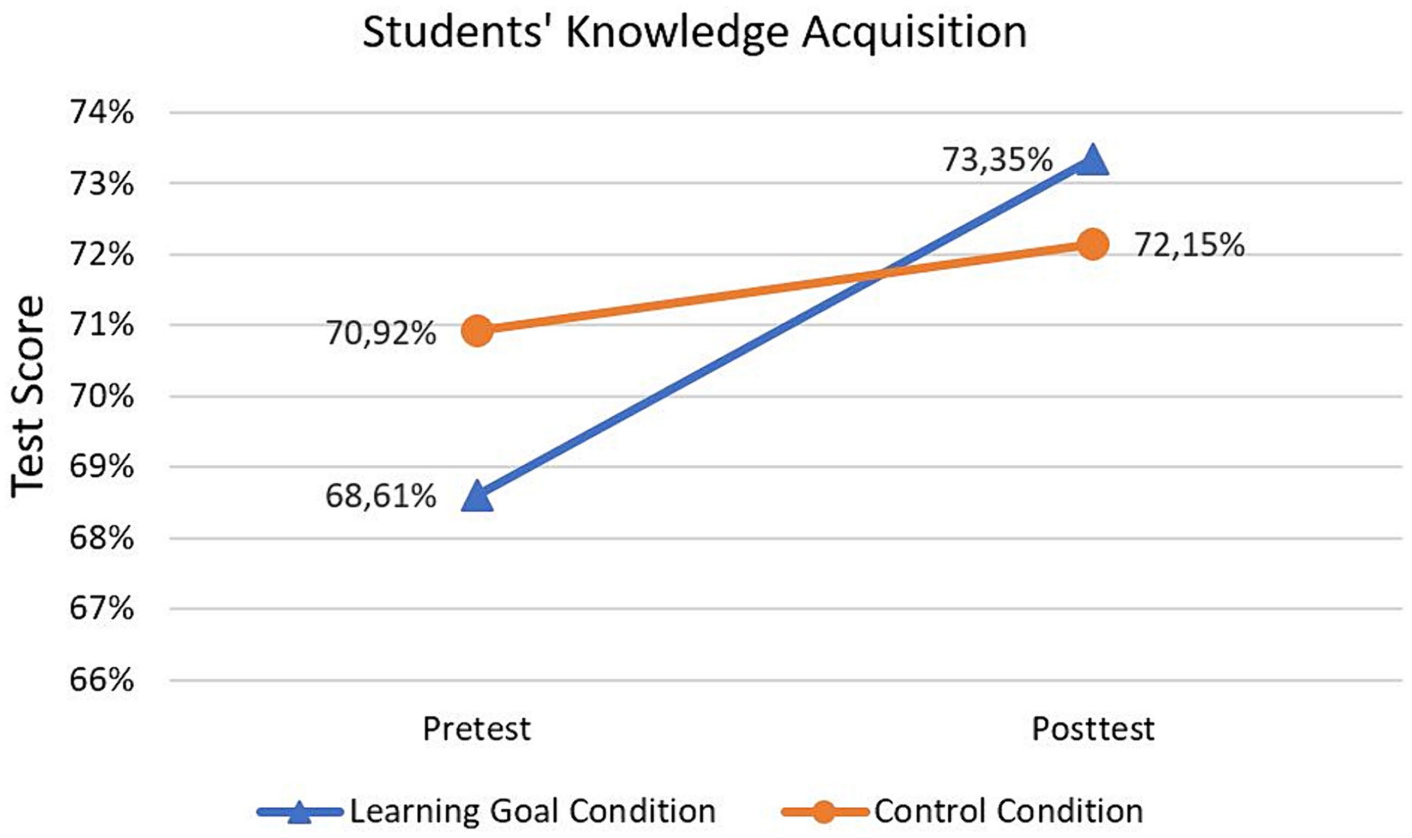
Means of students’ test score in pre- and posttest for the learning goal and control condition. The test score is calculated based on the percentage of correct answers out of 48 items. Therefore, the theoretical minimum and maximum is 0 and 100%, respectively.

With respect to RQ3 and H3, respectively, Figure 4 illustrates the development of students’ knowledge acquisition between pre- and posttest for the learning goal and control condition. Descriptively, students in both conditions displayed a slightly higher test score in the posttest than in the pretest. Moreover, the control group already exhibited a higher score in the pretest compared to the learning goal condition.

Regarding H3, a mixed ANOVA revealed that there was a significant interaction effect between time and condition, *F*(1, 143) = 5.28, *p* = 0.02, *η*^2^ = 0.04, indicating a small to medium effect (Cohen, 1988). However, there was no simple main effect of condition in the pretest (*η*^2^ = 0.014, *p* = 0.15) or posttest (*η*^2^ = 0.004, *p* = 0.46), which probably can be attributed to the crossover interaction (Sawilowsky and Sawilowsky, 2007). For this reason, we additionally conducted a *t*-Test with the difference in test scores between pre- and posttest as the dependent variable to compare the knowledge acquisition between the two groups. This yielded a significant difference with respect to the knowledge acquisition between the groups, with the difference in the test score being approximately 3,52% higher in the learning goal condition (95%CI [0.005, 0.065]), *t*(143) = 2.30, *p* = 0.01, Cohen’s *d* = 0.38. The simple main effect of time was significant in the learning goal condition, *F*(1, 71) = 23.76, *p* < 0.001, *η*^2^ = 0.25, but not in the control condition, *F*(1, 72) = 1.08, *p* = 0.30, *η*^2^ = 0.02.

## 5 Discussion

The StoG (Fischer et al., 2013) assumes that learners’ goals affect the configuration of learners’ internal collaboration scripts. However, to the best of our knowledge, this principle has not yet been tested directly empirically. Therefore, we investigated whether the induction of different achievement goals (Dweck and Leggett, 1988) affects the selection/change (H1) and sequential organization (H2) of the scriptlets that pre-service teachers select regarding a specific collaborative task and their subsequent knowledge acquisition (H3).

Regarding H1, we assumed that the induction of different kinds of achievement goals would affect the kinds of scriptlets learners select during collaboration. By means of the expert rating, the activities could be categorized as to whether they are typically associated with a learning goal orientation or performance goal orientation. Subsequently, for each of these two kinds of activities, we calculated a sum score for both pre- and posttest. Using these scores as dependent variable, we examined whether there were differences between the conditions. Based on the manipulation check, which only showed significant differences between the learning goal condition and the control condition, we narrowed down the analyses to just these two conditions and dropped the performance goal induction condition from the analyses. Our analyses did not indicate significant differences with respect to the change in the selected activities between the learning goal and control condition from pre- to posttest. However, the significant main effect of time indicates an influence of the collaborative case analysis regardless of a specific condition and might therefore be attributed to the task and the actual collaboration itself. Consequently, the hypothesis that the induction of a learning goal (as compared to no goal induction) would have an impact on the selection of scriptlets must be rejected, at least on the basis of the evidence regarding H1. In contrast, previous studies have suggested that learners engage in different activities (e.g., Pfister and Oehl, 2009) or strategies depending on their goals (e.g., Greisel et al., 2023). However, it is important to note that these studies examined the change or application of specific activities; in contrast, the present study involved categorizing a wider range of activities using expert ratings and investigating the changes within these categories. Therefore, on the one hand, this may mean that the change regarding the *kinds* of selected scriptlets is quite resistant to induced goals (at least with regard to learning goals). Moreover, a meta-analysis on goal induction (Noordzij et al., 2021) shows that to induce learning goals, it is important to relate this goal to a specific task (e.g., “While performing this task, it is your goal to… by …”). In comparison to this, the prompts used in our study may have been too vague. Thus, the goal induction may have been too weak at this point to have caused a change in the selection of kinds of scriptlets. On the other hand, these varying findings also raise the question of whether the assessments of the experts and learners might differ regarding which scriptlets are particularly useful for specific goals. For example, it is conceivable that some scriptlets are not considered particularly conducive to learning by students (e.g., based on their prior knowledge) and therefore are not used, whereas experts may categorize them as beneficial for learning (presumably based on their scientific knowledge). Future studies could therefore explore if and how the assessments of both diverge or, moreover, the basis on which learners come to use certain scriptlets in order to uncover the underlying mechanisms of students’ scriptlet selection.

With respect to H2, however, and in contrast to the selection of scriptlets, the results of the ENA showed significant differences in the configuration of the scriptlets between the learning goal induction condition and the control condition in the posttest. This means that the participants in the different conditions specified significantly different sequences of scriptlets they would apply in a new collaborative case analysis. This partially supports our hypothesis and can be seen as evidence in favor of the configuration principle, at least regarding the differing sequence (if not the type; see H1) of scriptlets. Through ENA, we can see that particular scriptlets were mentioned more frequently in a specific order by the groups. As described, learners with pronounced learning goals are particularly motivated to engage in learning because they focus on improving their competence (Heyman and Dweck, 1992). Given this context and the additional combination of this analysis with the expert ratings, it could be assumed that especially cognitive and elaborative learning activities (e.g., “explain case,” “compare solutions,” which were also associated with learning goals by the experts) are carried out when learning goals are induced. Such activities imply that learners construct new knowledge that goes beyond the scope of the existing learning materials (Chi and Wylie, 2014). In contrast, the ENA revealed that when no goals are induced, learners display a rather mixed picture with many different connections, which, nonetheless, also encompass activities related to learning goals. Yet, it can be noted that a part of these relatively strong connections among the activities “read theory,” “read case” and “read instructions” tends to reflect more superficial activities, with learners primarily engaged in merely receiving information. These activities, however, were not clearly assigned to any specific goal by the experts, likely because they could be interpreted as an inherent part of the instruction within the collaboration tool, independent of a specific goal of the learners. After all, further research is needed to examine which types and configurations of activities are most beneficial for a case analysis. However, the results of the ENA might suggest very goal-specific configurations which seem to largely be in line with results from research on achievement goals pointing to their context specificity (Daumiller, 2023).

To answer the second research question regarding the effects of goal induction on knowledge acquisition, we conducted a mixed ANOVA with the test score as dependent variable. Again, we excluded the performance goal condition due to the manipulation check, which is why the fourth hypothesis cannot be addressed. However, with respect to H3, we found a significant interaction effect for students’ test scores. In line with our hypothesis and prior research indicating favorable outcomes of learning goals (e.g., Hulleman et al., 2010; Payne et al., 2007), the induction of a learning goal had a stronger positive effect on students’ knowledge acquisition than the students carrying out the same tasks without goal induction. This highlights the significance of goal inductions not only on the configuration of learners’ internal scripts, but also on the knowledge they acquire through collaboration. This is remarkable, especially considering that the intervention in the current study was relatively simple, incorporating only relatively few goal-related prompts into the case analysis process. Although the present study identified only a small to moderate effect on knowledge acquisition, it is possible that more frequent and specific prompts could amplify this effect.

## 6 Limitations

Of course, this study does not come without limitations. First, it is important to note that in the instrument we used to assess students’ internal scripts, they selected from a range of activities, which means that they were not free in their choice of scriptlets, as certain activities were already suggested to them. It is quite conceivable that the students would also name other or further activities, possibly even more so if none were specified to them beforehand (Csanadi et al., 2021). Future studies could therefore include interviews, for example, to more validly capture script components and elicit their (re-)configuration more adequately (März et al., 2021).

Second, a type II error might have occurred. The effect sizes regarding the *selection* of scriptlets (H1) were very small (*η*^2^ < 0.01). In contrast, the sensitivity analysis indicated that the sample size was sufficient to detect effect sizes of at least Cohen’s *f* = 0.12 (*η*^2^ = 0.014). Thus, future studies should use larger samples to ensure greater statistical power.

In this context, it is also important to emphasize that in the ENA, the initial internal collaboration script of the learners was not taken into account and therefore only the differences in the subsequently reported activities can be determined, but not in comparison to the initial internal collaboration script. Thus, it would also be worthwhile considering a more process-oriented approach and, for example, monitoring activities in real time or using a thinking aloud approach in order to record the activities carried out as validly as possible.

Furthermore, only scriptlets were examined as internal script components in this study. Thus, our data did not allow for a separation of different internal script components beyond the scriptlet level. It is conceivable that learners already have had very heterogeneously elaborated scripts and therefore also responded differently to the external script (Kollar et al., 2007; Vogel et al., 2017). In this regard, the interaction of learners’ internal script levels and/or prior collaboration skills and goals might be an interesting research gap to look at in future studies.

A further potential limitation could be the presence of a selection bias. Although the study was incorporated into a university course of a large teacher education program, the decision to participate in data collection was voluntary and not rewarded. Consequently, the study may have attracted specifically students with pronounced learning goals and interest in their own competence development. Conversely, stronger performance goals could also have been induced because students felt that participation was still required as the study was part of the lecture. Nonetheless, it is important to point out that the present study found no significant differences in students’ learning or performance goal orientations between the groups that might account for the differences in the analyses.

Moreover, we only manipulated one type of goal in this study successfully. This also raises the question of how to effectively induce performance goals. For instance, it is conceivable that performance-related goals become particularly decisive when learners are more clearly aware that they are evaluated by others (Urdan and Mestas, 2006). For this reason, the asynchronous and non-graded case analysis in this study may not have sufficiently created this impression. Beyond performance and learning goals, there are many different kinds of achievement goals learners may have that could also be taken into account (e.g., avoidance goals; Daumiller, 2023). On top of that, research on achievement goals not only suggests that goals can be very situation-specific, but learners can also pursue multiple goals. This might also indicate that different and multiple goals might be particularly important in different collaboration scenarios (e.g., relational goals). Future research should therefore also include or control for further goals of the learners.

## 7 Conclusion

In sum, the results of the ENA indicate that learners’ internal collaboration scripts are configured differently depending on the induction of a learning goal. These results therefore support the StoG’s configuration principle that learners’ internal scripts are configured depending on learners’ current (situational) goals. This constitutes an important step in the empirical validation of the StoG and contributes to our understanding of the way internal collaboration scripts are configured in real situations.

### 7.1 Implications for theory and research

These findings offer several implications for theory and research. First, collaborative learners seem to be more likely to adapt only the sequential organization of their internal collaboration scriptlets to a goal than to add or omit scriptlets from different categories. We conclude that from the findings that indicate changes in the sequential organization, but not in the addition or removal of kinds of scriptlets. Due to learners’ prior internal collaboration scripts, they perhaps are more likely to modify the sequence of scriptlets in response to a goal induction (aligning with their prior internal collaboration scripts), rather than adding or omitting kinds of scriptlets they could be unfamiliar with. Future studies could therefore focus on how learners perceive and navigate the collaboration process and how prior internal collaboration scripts, external collaboration scripts, and goal-related prompts interact with each other. Moreover, this might also indicate the need to examine learners’ internal scripts on a rather fine-grained level.

Secondly, a closer look at the scriptlets also provided further insight into how certain activities are configured depending on a learning goal (e.g., as indicated by the sequential organization of the scriptlets “explain case” and “link knowledge”). In light of this, it also seems worthwhile to investigate the underlying mechanisms that determine why and how specific goals result in a specific reconfiguration in collaborative learning, that is, why learners (do not) apply specific activities in a specific manner.

### 7.2 Implications for practice

Furthermore, a number of practical implications can also be drawn from our findings. To begin with, teachers should consider the potential of the induction of goals. The manipulation of a goal resulting in differences in learners’ internal collaboration scripts and the subsequently higher knowledge acquisition highlights the importance of integrating prompts related to learning goals in CSCL to further tap into its potential for learning. For example, teachers may want to integrate prompts that target learning goals into the design of CSCL environments, possibly leading to script configurations and activities that are particularly conducive to student learning.

Consequently, there are also implications for teacher training programs. In teacher education, pre-service teachers should acquire knowledge about the goals of learners that particularly contribute to students’ collaborative learning, and how these can effectively be fostered through respective prompts within external scripts. This approach could also lead to more adaptive designs for computer-supported collaborative learning processes within higher education in general.

Eventually, this line of research offers the potential to elucidate the causal mechanisms by which the induction of goals impacts collaborative learning. In the future, this insight can help educators to apply new and improved strategies for effectively guiding activities to support student learning.

In conclusion, our study provides direct evidence for the SToG configuration principle and holds important implications regarding the design of CSCL-environments.

## Data Availability

The raw data supporting the conclusions of this article will be made available by the authors, without undue reservation.

## Appendix

**Table A1:** Overview of goal-related prompts for learning goal and performance goal condition.

Prompt learning goal	Prompt performance goal
*Acquisition of script knowledge*	
Please read the steps carefully *to understand how a case analysis can be carried out.*	Please read through the steps carefully *to be able to create particularly good case analyses.*
*Acquisition of theoretical knowledge*	
This knowledge is very important for you to *expand your skills*. The cognitive load theory/ICAP framework can be understood as part of the pedagogical-psychological professional knowledge of teachers. Reading the theoretical text and dealing with the case studies based on pedagogical-psychological theories represents an *important learning opportunity for you*. Various empirical studies in the field of psychology (e.g., Syring et al., 2015; Zumbach et al., 2008), as well as the experiences of practitioners suggest a positive effect of learning with authentic cases *on professional knowledge and later coping with problems in the classroom*. Therefore, take this opportunity to understand the cognitive load theory/ICAP framework as deeply as possible and *to be able to deal professionally* with problematic teaching situations.	This knowledge is very important in order to *achieve very good results in the state examination*. The cognitive load theory/ICAP model can be understood as part of the pedagogical-psychological professional knowledge of teachers. Reading the theoretical text and dealing with the case studies based on educational-psychological theories is *an important way for you to prepare for the exam so that you perform as well as possible in the state exam*. Various empirical studies in the field of psychology (e.g., Syring et al., 2015; Zumbach et al., 2008), as well as the experiences of practitioners suggest a positive effect of learning with authentic cases *on exam performance and subsequent performance in dealing with problems in the classroom. In addition, your performance will be assessed by the course tutors.* Therefore, take this opportunity to *demonstrate* your knowledge of the cognitive load theory/ICAP model and your ability to deal with problematic teaching situations *to the tutors and teachers of the course*.
*Case analysis*	
Please note that it is very important to complete the tasks conscientiously, *as this will allow you to deepen your knowledge and acquire important skills.*	Please note that it is very important to complete the tasks conscientiously, *as this will greatly increase your chances of being able to reproduce this knowledge later in exam situations and perform well.*

**Table A2:** List of activities with expert ratings.

read theory	search literature (L)
summarize	search peer mistake
underscore	show off complexity
list terms	have one’s will (P)
read instructions	compare quality (P)
relate theory and case (L)	assess competencies (P)
imagine application (L)	use technical terms
link knowledge (L)	surpass peer (P)
imagine examples (L)	ask peer (L)
relate with experience (L)	help peer (L)
think of meaning (L)	question comprehension (L)
examine evidence	imagine importance for performance (P)
scrutinize text (L)	compare with peer (P)
solve contradictions (L)	explain case (L)
compare solutions (L)	solve case
memorize subject matter (P)	identify case
repeat notes	set goal (L)
memorize terms (P)	describe case
memorize content (P)	read case
discuss case or theory (L)	read peer’s analysis
